# Use of acute phase proteins for the clinical assessment and management of canine leishmaniosis: general recommendations

**DOI:** 10.1186/s12917-018-1524-y

**Published:** 2018-06-20

**Authors:** J. J. Ceron, L. Pardo-Marin, M. Caldin, T. Furlanello, L. Solano-Gallego, F. Tecles, L. Bernal, G. Baneth, S. Martinez-Subiela

**Affiliations:** 1 Interdisciplinary Laboratory of Clinical Analysis (Interlab-UMU), Campus of Excellence Mare Nostrum, University of Murcia, 30100 Espinardo, Murcia, Spain; 2 San Marco Veterinary Clinic, Veggiano, Padova, Italy; 3 Animal Medicine and Surgery Department, Universitat Autònoma de Barcelona, Cerdanyola del Valles, Spain; 4 Koret School of Veterinary Medicine, Hebrew University, Rehovot, Israel

**Keywords:** Canine leishmaniosis, Acute phase proteins, Staging

## Abstract

**Background:**

Dogs with canine leishmaniosis (CanL) due to *Leishmania infantum* can show a wide spectrum of clinical and clinicopathological findings at the time of diagnosis. The aim of this paper is to describe the possible application of acute phase proteins (APPs) for the characterization and management of this disease, based on previously published information on the utility of APPs in CanL and the experience of the authors in using APPs as analytes in the profiling of canine diseases.

**Main body:**

Dogs diagnosed with *L. infantum* infection by serology, polymerase chain reaction, cytological or histopathological identification, can be divided into three groups based on their clinical condition at physical examination and their APPs concentrations: Group 1: dogs with no clinical signs on physical examination and APPs in reference range; Group 2: dogs with changes in APPs but no clinical signs on physical examination; Group 3: dogs with clinical signs and changes in APPs**.** This report describes the main characteristics of each group as well as its association with the clinical classification schemes of CanL.

**Conclusion:**

APPs concentration can be a useful clinical tool to characterize and manage CanL.

## Background

Acute phase proteins (APPs) are important components of the innate immune system response that change in concentration when inflammation occurs [1]. They are considered as sensitive markers of the activation of the immune system and therefore of the presence of an active inflammation. Knowledge on their changes in a variety of diseases and their usefulness as markers, as well as the increase in the availability of different assays for their measurements, has produced useful applications in the companion animal routine clinical practice [2, 3].

Canine leishmaniosis (CanL) caused by *Leishmania* spp*.* is a complex and common zoonotic disease in large areas of the world which can be life-threatening to dogs and humans. It affects several million domestic dogs in countries on both shores of Atlantic Ocean, mainly in Europe and Central and South America, but also in Africa and Asia as well [4]. CanL is diagnosed by tests such as serology, polymerase chain reaction (PCR) or observation of the parasite in tissues such as cutaneous lesions, lymph nodes or bone marrow [5].

Two main staging systems of CanL have been proposed in the last decade. The LeishVet group has classified four stages of disease in dogs with clinical signs [5], whereas the Canine Leishmaniasis Working Group (CLWG) has proposed another system which includes not only sick dogs, but also those considered exposed or infected with no clinical manifestations of the disease [6].

Studies on naturally-occurring and experimental CanL have demonstrated that there is an increase in positive APPs such as C-reactive protein (CRP), haptoglobin (Hp) and ferritin and a decrease in negative APPs such as albumin, paraoxonase 1 (PON1) or apolipoprotein 1 (Apo-A1) during various stages of the disease [7–10]. The clinical application of APPs in dogs with this disease is often focused on the monitoring of treatment, since a decrease in positive APPs and an increase in negative APPs is associated with an adequate response to treatment, as also demonstrated in humans with visceral leishmaniosis [11–13].

In our experience, during years of using APPs as analytes in the profiling of canine diseases, we have observed that CRP and ferritin can be potentially applied also for the classification and management of the disease, both at the time of initial diagnosis or in previously diagnosed dogs, during and after treatment. This report describes and provides some general recommendations about the application of acute phase proteins (APPs) for the characterization and management of CanL, based on previously published studies [7–9, 13–15] and the experience of the authors in using APPs in this disease. The authors propose that dogs diagnosed with *L. infantum* infection by serology, PCR, cytological or histopathologic identification can be divided into three groups based on their clinical condition at physical examination and their APP concentrations. This report describes the main characteristics of each group as well as its possible association with the classification schemes of CanL (Table 1).

**Table 1:** Characteristics of different groups of dogs infected with *Leishmania infantum* based on their APP response

	Characteristics		Types of dog that fit each category	Correspondence with guidelines	
	CRP and ferritin	Clinical signs at physical exam		LeishVet	CLWG
Group 1	In reference range	No	Infected but resistant to the development of clinical leishmaniosis. Dogs that have responded to treatment. Infected but no APR yet developed.	Not applicable	Stage A (exposed) Stage B (infected)
Group 2	Moderate increase in one or both of them	No	Expected to exhibit clinical signs in the future. Have other conditions that increase APPs (infections, neoplasms or trauma).	Not applicable	Stage A (exposed) Stage B (infected) Stage C (sick dogs)
Group 3(a)	Moderate increase in one or both of them	Yes	Dogs with clinical leishmaniosis (mild to moderate disease).	Stages I and II (mild to moderate disease)	Stage C (sick dogs)
Group 3(b)	Major increase in one or both of them	Yes	Dogs with severe clinical leishmaniosis with immunomediated complications.	Stages III and IV (severe to very severe disease)	Stage D (severely sick dogs)

## Main body

### Group 1. Dogs with no clinical signs of CanL on physical examination and APPs in reference range

Dogs in this group do not have clinical signs related to CanL found on physical examination and/or clinicopathological abnormalities [5–7] and have CRP and ferritin values within the laboratory reference ranges. This group of dogs would comprise of:

- Dogs which are infected but resistant to the development of clinical leishmaniosis due to an adequate cellular immune response. Previous studies have concluded that dogs in this status should be monitored but not necessarily treated [16].
- Dogs that have responded adequately to treatment for the disease. It has been reported that CRP and ferritin concentrations within the reference ranges found in treated dogs are usually associated with the absence of clinical signs and good response to treatment, despite the presence of abnormalities in the concentration of total proteins or globulins [8, 9, 13]. This is because CRP and ferritin changes tend to normalize when clinical signs of the disease disappear. This may occur earlier than the return of serology to negative levels and the decrease in globulins into reference ranges [8, 13].
  These dogs could have persistent proteinuria due to kidney pathology that may have occurred when the disease was active and therefore, in this situation, they should be treated following the IRIS guidelines for proteinuria treatment [5, 17].
- Dogs that have been recently infected and have not yet developed an acute phase response (APR), as described in an experimental infection [8].

Group 1 would correspond with the CLWG stage A (exposed dogs) or stage B (infected dogs).

### Group 2. Dogs with no clinical signs of CanL on physical examination and changes in APPs

Dogs in group 2 have moderate increases (usually not more than 5 times higher than the reference range) in CRP and/or ferritin. However they do not have clinical signs related with CanL on physical examination, and may or may not have hematological abnormalities found on complete blood count (CBC) or alterations of the serum biochemical profile.

Dogs in group 2 may often go on to developing clinical signs of CanL, as demonstrated in an experimental infection [8]. In previous studies, a rise in APPs was noted two months before the appearance of clinical signs [8, 9]. This could be explained by the fact that APPs are components of the innate immune response representing the first line of defense against infecting organisms. The infected host may respond rapidly to infection as expressed by rapid changes in the APP concentrations. Therefore, APPs may increase even prior to the appearance of clinical signs of leishmaniosis. From a clinical point of view, an increase in APPs in a dog positive by serology and/or PCR to *Leishmania* but apparently healthy, can forecast the appearance of clinical signs in the future.

Dogs that go on to developing clinical disease usually have a particular APP profile with moderate increases in CRP and ferritin characterized by concentrations that are usually not more than 5 times higher than the reference range, as reported in experimental studies [8, 9]. This profile can be considered characteristic since CRP, as a major APP, usually shows increases of higher magnitude than ferritin in inflammatory conditions.

Dogs in group 2 can have changes in CBC and biochemical profiles and, in these cases, they could correspond with CLWG stage C (diseased dogs). But a fraction of the dogs in group 2 may not have changes in CBC, biochemical profile (if APPs are not included) and urinalysis and would correspond with CLWG stage A (exposed dogs) or stage B (infected dogs). In these cases, the increase in APPs would indicate a risk of developing the disease in the future.

It should be pointed out that not all increases in APPs in dogs with *Leishmania* infection are always due to a worsening of the infection. These dogs may have other reasons for the increase in these proteins, like conditions due to other causes such as other infections, neoplasia or trauma [3, 18, 19]. Dogs in this group should be carefully evaluated and be treated against the underlying cause of APP increase.

### Group 3. Dogs with clinical signs of CanL on physical examination and changes in APPs

Dogs in this group have clinical signs compatible with CanL on physical examination and APPs changes. These dogs would correspond to the spectrum of LeishVet stages 1 to 4 and to CLWG groups C and D [5, 6]. They can be additionally divided according to their APP concentrations into:

- Group 3a. Dogs that have a moderate increase in CRP and ferritin (usually not more than 5 fold), similarly to those previously reported in humans [20], and moderate to severe clinical signs. Dogs in group 3a suffer from clinical leishmaniosis and should therefore be treated with the anti-leishmanial drugs currently recommended [4, 5, 21]. This would include dogs in LeishVet stages 1 to 2 and CLWG stage C (clinically diseased).
- Group 3b. Dogs that have major increases in CRP and ferritin (usually more than 5 fold). In our experience, dogs in this condition usually have evident immune-mediated complications such as uveitis, severe joint disease or immune-mediated cytopenias. These conditions represent a severe form of CanL with an exacerbation of the immune response which could be devastating. This would include dogs in LeishVet stages 3 and 4 and CLWG stage D (severely clinically diseased).

Some dogs of group 3b may suffer from kidney failure with considerable increases in serum creatinine levels. In such cases of severe kidney disease, CRP and ferritin usually do not increase in such a high magnitude and could have values corresponding with group 3a, and CRP can even in some cases be in the normal reference values [14, 22]. Conversely, in some cases dogs with moderate clinical signs and no evident immune-mediated complications can show CRP and ferritin values of high magnitude (more than 5 fold), these cases would represent probably a transition from the group 3a to 3b.

An association between ferritin concentrations and immune-mediated disease has been described in humans with diseases such as lupus erythematosus or rheumatoid arthritis, where the magnitude of increase in ferritin parallels the severity of clinical signs [23–25]. Likewise, dogs with immune mediated polyarthritis or immune mediated hemolytic anemia have high CRP values [26–29].

### Strengths and limitations of the recommendations

Currently the diagnosis and management of CanL and the follow-up of treated dogs combines clinicopathological tests such as CBC, serum biochemistry profile and urinalysis with serological and parasitological tests. The measurement of APP concentrations represents an additional dimension to the diagnostic and follow up processes, as APPs may increase along the dog’s progression from sub-clinical infection into clinical disease, or decrease during treatment even before the improvement in other clinical pathology parameters and clinical signs, signaling the advent of response to drug therapy [8, 9, 13].

It should be pointed out that the correspondence of the groups in this report to other existing clinical staging systems should be taken with caution, since in some cases, such as stage C of the CLWG, an overlap can occur. This scheme is not proposed as a substitute to the existing staging systems, it is a division of dogs to groups according to their APP responses and a description of the potential benefits that this division could bring. Therefore it could be an ancillary tool to help the veterinary clinician in the decision making process related to the management of CanL cases. For example, in group 1, treatment against CanL may not be needed, although a close monitoring should be made. In group 2, dogs may develop clinical signs at physical exam in the future, therefore a close follow-up and eventually therapy could be recommended. In group 3 therapy would be recommended, and in particular the group 3b could have the potential benefit of immunosupressive therapy. This is only a recommendation for the management of clinical cases and should be interpreted with caution since, in many countries, treatment of infected dogs is mandatory, independently on the presence or absence of clinical or laboratory abnormalities, due to public health reasons.

Although this review refers mostly to CRP and ferritin, changing patterns are found also in other APPs measured in canine *L. infantum* infection, for example in PON1. Usually there are no changes in PON1 activity in group 1, moderate decreases in group 2 and more pronounced decreases in group 3 [9].

Despite the usefulness of APPs in the evaluation of dogs with CanL, it should be considered that at this time there is insufficient data on certain questions such as the possible use of a cut-off in CRP and ferritin values in order to stop the therapy due to resolution of the disease. In addition, the fold increases proposed to consider moderate or major increases are approximate and should be more precisely defined in studies with a large population. These questions and others will require further studies to determine the usefulness of these APPs in making clinically-related decisions. Another important point is that, although the measurement of APPs can be helpful for the clinician in determining the clinical status of a previously diagnosed dog, specific tests for the diagnosis of CanL or for evaluation of kidney disease status cannot be substituted by determining the APP levels, and APPs are therefore only used as ancillary tests. In addition, the data presented here is based on dogs infected with *L. infantum* but it is not known if infection of dogs with other *Leishmania* spp. could present other scenarios and differences in APP responses. Also, since CanL is a complex disease, there could be situations that may not fit exactly into any of the 3 groups. For example, dogs with only papular dermatitis, despite having this clinical sign at physical examination usually do not show increases in APPs. In our opinion these dogs could be considered as more related to Group 1, since they have an adequate cellular immune response that makes them resistant to the development of a more severe form of clinical leishmaniosis [30].

Furthermore, increases in APP concentrations would not always indicate the transition from sub-clinical infection to clinical leishmaniosis. This is due to the low specificity of these analytes, because many other conditions can increase their concentration [31, 32]. Increase in positive APPs would in any case warrant the need for a careful evaluation of the infected dog. Also, there are some situations such as glucocorticoid treatment where the response of some APPs such as CRP can be inhibited [33].

Therefore, long-term studies would be beneficial in strengthening and refining the value of measurement of APPs levels in CanL and in further defining the APP levels in different scenarios of sub-clinical infection, disease and post-treatment responses.

## Conclusions

The authors propose that dogs infected with *L. infantum* can be divided into three groups based on their clinical condition at physical examination and their APP concentrations: Group 1: dogs with no clinical signs on physical examination and APPs in reference range; Group 2: dogs with no clinical signs on physical examination but changes in APPs; and Group 3: dogs with clinical signs and changes in APPs**.** In addition, monitoring of APP levels could help to identify animals that are in transition from one stage to another, such as from sub-clinical infection into clinical disease, or even signaling the advent of response to drug therapy.

Overall this division can help in taking the most appropriate clinical decisions in the management of CanL cases.

## Abbreviations

APO A1: Apolipoprotein A1
APPs: Acute phase proteins
APR: Acute phase response
CanL: Canine Leishmaniosis
CBC: Complete blood count.
CLWG: Canine leishmaniasis working group
CRP: C-reactive protein
PCR: Polymerase chain reaction
PON1: Paraoxonase 1
